# Global dynamics for discrete-time analog of viral infection model with nonlinear incidence and CTL immune response

**DOI:** 10.1186/s13662-016-0862-y

**Published:** 2016-05-23

**Authors:** Jianpeng Wang, Zhidong Teng, Hui Miao

**Affiliations:** College of Mathematics and Systems Science, Xinjiang University, Urumqi, 830046 People’s Republic of China

**Keywords:** 37M99, 39A11, 92D30, viral infection model, CTL immune response, NSFD scheme, basic reproduction number, local and global stability

## Abstract

In this paper, a discrete-time analog of a viral infection model with nonlinear incidence and CTL immune response is established by using the Micken non-standard finite difference scheme. The two basic reproduction numbers $R_{0}$ and $R_{1}$ are defined. The basic properties on the positivity and boundedness of solutions and the existence of the virus-free, the no-immune, and the infected equilibria are established. By using the Lyapunov functions and linearization methods, the global stability of the equilibria for the model is established. That is, when $R_{0}\leq1$ then the virus-free equilibrium is globally asymptotically stable, and under the additional assumption $(A_{4})$ when $R_{0}>1$ and $R_{1}\leq1$ then the no-immune equilibrium is globally asymptotically stable and when $R_{0}>1$ and $R_{1}>1$ then the infected equilibrium is globally asymptotically stable. Furthermore, the numerical simulations show that even if assumption $(A_{4})$ does not hold, the no-immune equilibrium and the infected equilibrium also may be globally asymptotically stable.

## Introduction

As is well known, viruses have caused the abundant types of epidemics and are alive almost everywhere on Earth, infecting people, animals, plants, and so on. There are a large number of diseases, which are caused by viruses for example: influenza, hepatitis, HIV, AIDS, SARS, Ebola, MERS. Therefore, it is important to study viral infection, which can supply theoretical evidence for controlling a disease to break out. In the past years, many authors have studied continuous time viral infection models which are described by the differential equations. See, for example, [1–28] and the references cited therein.

In [1], Hattaf *et al.* proposed the following continuous time viral infection model:

1 $$ \textstyle\begin{cases} \frac{\mathrm {d}x(t)}{\mathrm {d}t}=\lambda-d x-f(x,y,v)v,\\ \frac{\mathrm {d}y(t)}{\mathrm {d}t}=f(x,y,v)v-ay,\\ \frac{\mathrm {d}v(t)}{\mathrm {d}t}=ky-uv, \end{cases} $$

where *x*, *y*, and *v* denote the densities of uninfected cells, infected cells and virus cells, respectively, *λ* is the rate of production of uninfected cells, *d* is the death rate of uninfected cells, $f(x,y,v)$ is the rate of uninfected cells to become infected by virus, *a* is the rate of disappearance of infected cells, *k* is the rate that virus produces by infected cells, and *u* is the rate of virus died. The dynamic behaviors of the model are studied. Although model (1) is simple, model (1) is very important in viral epidemiology, which can show ample viral behaviors. Then, based on continuous model (1), Shi and Dong in [22] proposed a discrete-time analog for the special case $f(x,y,v)=\beta x$ of model (1) by using Micken’s non-standard finite difference (NSFD) scheme. The authors studied the local and global stability of the equilibria and the permanence of the model. In [26], Hattaf and Yousfi proposed the following discrete-time analog directly for model (1) by using the NSFD scheme:

2 $$ \textstyle\begin{cases} x_{n+1}= x_{n}+h(\lambda-d x_{n+1}-f(x_{n+1},y_{n},v_{n})v_{n}),\\ y_{n+1}= y_{n}+h(f(x_{n+1},y_{n},v_{n})v_{n}-ay_{n+1}),\\ v_{n+1}= v_{n}+h(ky_{n+1}-uv_{n+1}), \end{cases} $$

where $n\in N$, and *N* denotes the set of all non-negative integers. The global asymptotic stability of the disease-free equilibrium and the chronic infection equilibrium is established by constructing the suitable Lyapunov functions. In [28], the authors extended model (2) to the delayed case. By using the method of Lyapunov functions, the authors established the global asymptotic stability of the disease-free equilibrium and the chronic infection equilibrium with no restriction on the time-step size.

In general, our target is to eliminate and control the virus and infected cells. For all this, many authors have noted that the immune response takes great effect to eliminate and control the virus and infected cells because CTL (cytotoxic T lymphocyte) cells affect the virus load. Therefore, a four dimension continuous time virus dynamical model with Beddington-DeAngelis incidence rate and CTL immune response was studied by Wang, Tao and Song in [2]. The model proposed is as follows:

3 $$ \textstyle\begin{cases} \frac{\mathrm {d}x(t)}{\mathrm {d}t}=\lambda-d x-\frac{\beta xv}{1+mx+nv},\\ \frac{\mathrm {d}y(t)}{\mathrm {d}t}=\frac{\beta xv}{1+mx+nv}-ay-pyz,\\ \frac{\mathrm {d}v(t)}{\mathrm {d}t}=ky-uv,\\ \frac{\mathrm {d}z(t)}{\mathrm {d}t}=cyz-bz. \end{cases} $$

The authors established the global stability of the disease-free equilibrium, the immune-free equilibrium, and the endemic equilibrium.

Motivated by the above works, in this paper we consider a discrete-time analog of a class of continuous time virus dynamical models with nonlinear incidence and CTL immune response which is established by using NSFD scheme. The model is proposed in the following form:

4 $$ \textstyle\begin{cases} \frac{x_{n+1}-x_{n}}{\phi}=\lambda -d x_{n+1}-f(x_{n+1},y_{n},v_{n})v_{n},\\ \frac{y_{n+1}-y_{n}}{\phi }=f(x_{n+1},y_{n},v_{n})v_{n}-ay_{n+1}-py_{n+1}z_{n+1},\\ \frac{v_{n+1}-v_{n}}{\phi}=ky_{n+1}-uv_{n+1},\\ \frac{z_{n+1}-z_{n}}{\phi}=cy_{n+1}z_{n+1}-bz_{n+1}, \end{cases} $$

where $x_{n}$, $y_{n}$, $v_{n}$ and $z_{n}$ denote the densities of uninfected cells, infected cells, virus cells, and CTL cells at time *n*, respectively. The parameters *λ*, *d*, *a*, *k*, and *u* have the same biological meanings as in model (1), *p* is the removed rate for the infected cells by the CTL immune response, *c* is the proliferated rate for the CTL cells by contact with infected cells, *b* is the disappearance rate for the CTL cells, and the function *ϕ* is a denominator function (see [29, 30]), which is defined by

$$\phi=\phi(h)=\frac{e^{d h}-1}{d }. $$

It is well known that the non-standard scheme satisfies the following important rules: the standard denominator *h* in standard discrete derivative is replaced by a denominator function $0<\phi(h)<1$, where $\phi(h)=h+o(h^{2})$ and *h* is the time-step size of numerical integration, and the nonlinear terms are approximated in a nonlocal way using more than one mesh point (see [31, 32]).

Particularly, when $f(x,y,v)=\frac{\beta x}{1+mx+nv}$, we can get the corresponding discrete-time analog of continuous model (3) as follows:

5 $$ \textstyle\begin{cases} \frac{x_{n+1}-x_{n}}{\phi} =\lambda-d x_{n+1}-\frac{\beta x_{n+1}}{1+mx_{n+1}+nv_{n}}v_{n},\\ \frac{y_{n+1}-y_{n}}{\phi} =\frac{\beta x_{n+1}}{1+mx_{n+1}+nv_{n}}v_{n}-ay_{n+1}-py_{n+1}z_{n+1},\\ \frac{v_{n+1}-v_{n}}{\phi} =ky_{n+1}-uv_{n+1},\\ \frac{z_{n+1}-z_{n}}{\phi} =cy_{n+1}z_{n+1}-bz_{n+1}. \end{cases} $$

In this paper, our main purpose is to study the threshold dynamics of model (4). The two basic reproduction numbers $R_{0}$ and $R_{1}$ are defined. The basic properties on the positivity and boundedness of solutions and the existence of the virus-free equilibrium, the no-immune equilibrium and the infected equilibrium are established. By using the Lyapunov functions and linearization methods, we will establish a series of criteria to ensure the stability of the equilibria for model (4). That is, we will prove that when $R_{0}\leq1$ then model (4) only has the virus-free equilibrium and it is globally asymptotically stable, when $R_{0}>1$ and $R_{1}\leq1$ then model (4) has only the virus-free and the no-immune equilibria, the virus-free equilibrium is unstable and under the additional assumption $(A_{4})$ (see Section 3) the no-immune equilibrium is globally asymptotically stable, and lastly when $R_{0}>1$ and $R_{1}>1$ then model (4) has three equilibria: the virus-free equilibrium, the no-immune equilibrium, and the infected equilibrium; the virus-free and the no-immune equilibria are unstable and under the additional assumption $(A_{4})$ the infected equilibrium is globally asymptotically stable. Furthermore, numerical simulations are given. It is shown that even if assumption $(A_{4})$ does not hold, the no-immune equilibrium may be globally asymptotically stable only when $R_{0}>1$ and $R_{1}<1$, and the infected equilibrium may be globally asymptotically stable only when $R_{0}>1$ and $R_{1}>1$.

This paper is organized as follows. In Section 2, we will first introduce some assumptions for nonlinear incidence function $f(x,y,v)$. Next, we will state and prove some basic results on the existence, uniqueness, positivity and ultimate boundedness of solutions with positive initial conditions for model (4). Furthermore, the existence of the virus-free, the no-immune, and the infected equilibria also is obtained. The stability of the virus-free, the no-immune, and the infected equilibria is presented in Section 3. The numerical simulations are presented in Section 4. Lastly, some concluding remarks are presented in Section 5.

## Preliminaries

As the epidemiological background of model (4), we assume that any solution $(x_{n},y_{n},v_{n},z_{n})$ of model (4) satisfies the following initial condition:

6 $$ x_{0}>0,\quad\quad y_{0}>0,\quad\quad v_{0}>0,\quad\quad z_{0}>0. $$

We also require that the function $f(x,y,z)$ satisfies the following assumptions:

$(A_{1})$
$f(0,y,v)=0$ for all $y\geq0$ and $v\geq0$,
$(A_{2})$
$\frac{\partial f(x,y,v)}{\partial x}>0$ for all $x>0$, $y\geq 0$ and $v\geq0$,
$(A_{3})$
$\frac{\partial f(x,y,v)}{\partial y}\leq0$ and $\frac {\partial f(x,y,v)}{\partial v}\leq0$ for all $x\geq0$, $y\geq0$ and $v\geq0$.

Specially, when $f(x,y,v)=\frac{\beta x}{1+mx+nv}$ and $f(x,y,v)=\frac {\beta x}{1+nv^{q}}$, where $\beta>0$, $m\geq0$, $q\geq0$, and $n\geq 0$ are constants, by simple calculation we know that such $f(x,y,v)$ satisfies the above assumptions $(A_{1})$-$(A_{3})$.

### Lemma 1

*Let* $(A_{1})$ *and* $(A_{2})$ *hold*. *Then the solution* $(x_{n},y_{n},v_{n},z_{n})$ *of model* (4) *with initial value* (6) *exists uniquely and is positive for all* $n\in N$. *In addition*, $0< y_{n}<\frac{1+b\phi}{c\phi}$ *for* $n=1,2, \ldots$ .

### Proof

We know that model (4) is equivalent to the following form:

7 $$ \textstyle\begin{cases} x_{n+1} =\frac{1}{1+\phi d}(x_{n}+\phi(\lambda -f(x_{n+1},y_{n},v_{n})v_{n})),\\ y_{n+1} =\frac{y_{n}+\phi f(x_{n+1},y_{n},v_{n})v_{n}}{1+\phi (a+pz_{n+1})},\\ v_{n+1} =\frac{v_{n}+\phi ky_{n+1}}{1+\phi u},\\ z_{n+1} =\frac{z_{n}}{1+\phi(b-cy_{n+1})}. \end{cases} $$

When $n=0$, we prove that $(x_{1}, y_{1}, v_{1}, z_{1})$ exists uniquely and is positive.

We first consider $x_{1}$. According to the first equation of model (7), we have

$$\varphi(x_{1})\triangleq x_{1}+\phi \bigl[d x_{1}+f(x_{1},y_{0},v_{0})v_{0}- \lambda\bigr]-x_{0}=0. $$

Owing to $\varphi(0)=-x_{0}-\phi\lambda<0$, $\lim_{x_{1}\to\infty}\varphi (x_{1})=\infty$ and from $(A_{2})$,

$$\varphi'(x_{1})=1+\phi \biggl[d+\frac{\partial f}{\partial x_{1}}(x_{1},y_{0},v_{0})v_{0} \biggr]>0. $$

Hence, there is a unique $x_{1}>0$ such that $x_{1}=x_{0}+\phi[\lambda-d x_{1}-f(x_{1},y_{0},v_{0})v_{0}]$.

Next, we consider $z_{1}$. According to the second and fourth equations of model (7), we have

8 $$ z_{1}= z_{0}+\phi \biggl[c \frac{y_{0}+\phi f(x_{1},y_{0},v_{0})v_{0}}{1+\phi(a+pz_{1})}z_{1}-bz_{1} \biggr]. $$

Let

$$ \begin{aligned} \varphi(z_{1})&\triangleq\phi p(1+\phi b)z_{1}^{2}+\bigl(1+\phi\bigl[a-p z_{0}-cy_{0} \\ &\quad{}-\phi cv_{0}f(x_{1},y_{0},v_{0})+b(1+ \phi a)\bigr]\bigr)z_{1}-z_{0}(1+\phi a). \end{aligned} $$

This is a quadratic function. Since $\varphi(0)=-z_{0}(1+\phi a)<0$ and $\lim_{z_{1}\to\infty}\varphi (z_{1})=\infty$, there is a unique $z_{1}>0$ such that $\varphi(z_{1})=0$. That is, (8) holds.

In the following, we consider $y_{1}$. According to the second and last equations of model (7), we have

9 $$ y_{1}=y_{0}+\phi \biggl[f(x_{1},y_{0},v_{0})v_{0}-ay_{1}-py_{1} \frac {z_{0}}{1+\phi(b-cy_{1})} \biggr]. $$

Let

$$ \begin{aligned} \varphi(y_{1})&\triangleq\phi\bigl[c(1+ \phi a)y^{2}_{1}-\bigl(b+cy_{0}+\phi cf(x_{1},y_{0},v_{0})v_{0}+a \\ &\quad{}+\phi ab+pz_{0}\bigr)y_{1}+y_{0}b+(1+\phi b)f(x_{1},y_{0},v_{0})v_{0} \bigr]+y_{0}-y_{1}. \end{aligned} $$

Owing to $z_{1}>0$, from the last equation of model (7) we have $y_{1}<\frac{1+\phi b}{\phi c}$. Then we have

$$\begin{aligned}& \varphi(0)=y_{0}+\phi\bigl[y_{0}b+(1+\phi b)f(x_{1},y_{0},v_{0})v_{0}\bigr]>0, \\& \varphi\biggl(\frac{1+\phi b}{\phi c}\biggr)=-\frac{pz_{0}+\phi pbz_{0}}{c}< 0. \end{aligned}$$

Since $\varphi(y_{1})$ is a quadratic function, there is a unique $y_{1} \in(0,\frac{1+\phi b}{\phi c})$ such that $\varphi(y_{1})=0$. That is, (9) holds.

Finally, we consider $v_{1}$. According to the third equation of model (7), we have $v_{1}=\frac{v_{0}+\phi ky_{1}}{1+\phi u}$. Hence, we know that $v_{1}$ uniquely exists and is positive. Therefore, $(x_{1},y_{1},v_{1},z_{1})$ exists uniquely and is positive.

When $n=1$, by a similar argument to the above, we can prove that $(x_{2}, y_{2}, v_{2}, z_{2})$ exists uniquely and is positive. Owing to $z_{2}>0$, we also have $y_{2}<\frac{1+\phi b}{\phi c}$. Using the mathematical induction, for any $n\geq0$, we know that $(x_{n},y_{n},v_{n},z_{n})$ exists uniquely and is positive. Furthermore, we also have $y_{n}<\frac{1+\phi b}{\phi c}$. This completes the proof. □

Let us consider the region

$$\Gamma=\biggl\{ (x,y,v,z): 0< x,y,v,z\leq\frac{\lambda}{\xi}\biggr\} , $$

where $\xi=\min\{d,\frac{a}{2},u,b\}$. We have the following result.

### Lemma 2

*Any solution* $(x_{n},y_{n},v_{n},z_{n})$ *of model* (4) *with initial condition* (6) *converges on* Γ *as* $n\to\infty$, *and* Γ *is positive invariable for model* (4).

### Proof

Define a sequence $M_{n}$ as follows:

$$M_{n}=x_{n}+y_{n}+\frac{a}{2k}v_{n}+ \frac{p}{c}z_{n}. $$

We have

$$ \begin{aligned} M_{n+1}&= x_{n+1}+y_{n+1}+ \frac{a}{2k}v_{n+1}+\frac{p}{c}z_{n+1} \\ &= x_{n}+y_{n}+\frac{a}{2k}v_{n}+ \frac{p}{c}z_{n}+\phi[\lambda -d x_{n+1}-ay_{n+1} -py_{n+1}z_{n+1}] \\ &\quad{}+\phi \biggl[\frac{a}{2}y_{n+1}-\frac{au}{2k}v_{n+1}+py_{n+1}z_{n+1} -\frac{pb}{c}z_{n+1} \biggr] \\ &= M_{n}+\phi \biggl[\lambda-d x_{n+1}-\frac{a}{2}y_{n+1}- \frac{au}{2k}v_{n+1} -\frac{pb}{c}z_{n+1} \biggr] \\ &\leq M_{n}+\phi[\lambda-\xi M_{n+1}]. \end{aligned} $$

Hence,

10 $$ M_{n+1}\leq\frac{1}{1+\phi\xi}M_{n}+ \frac{\phi\lambda}{1+\phi \xi}. $$

By using the induction, we have

$$M_{n}\leq\biggl(\frac{1}{1+\phi\xi}\biggr)^{n}M_{0}+ \frac{\lambda}{\xi} \biggl[1-\biggl(\frac{1}{1+\phi\xi}\biggr)^{n} \biggr]. $$

Consequently, $\limsup_{n \rightarrow\infty} M_{n}\leq\frac{\lambda}{\xi}$. Owing to the positivity of solution $({x_{n}},{y_{n}},{v_{n}},{z_{n}})$, we see that $({x_{n}},{y_{n}},{v_{n}},{z_{n}})$ converges on Γ as $n\to \infty$. Furthermore, from Lemma 1 and (10), we easily see that Γ is positive invariable for model (4). This completes the proof. □

The basic reproductive numbers for model (4) are given by

$$R_{0}=\frac{kf(\frac{\lambda}{d},0,0)}{au},\quad \quad R_{1}=\frac{c}{b}y^{*}_{1}, $$

where $y^{*}_{1}$ is given in the following conclusion (ii) of Lemma 3. $R_{0}$ is defined as the average number of secondary infected cells generated by a single infected cell put in an uninfected cell (or free virus) population, $R_{1}$ is defined as the average number of killed infected cells by a single CTL cell contacting the infected cells. Based on these basic reproductive numbers, we give the following lemma.

### Lemma 3

*Let* $(A_{1})$-$(A_{3})$ *hold*.

(i) *Model* (4) *always has a virus*-*free equilibrium* $E_{0}(\frac {\lambda}{d},0,0,0)$.
(ii) *If* $R_{0}\leq1$, *then model* (4) *has only a virus*-*free equilibrium* $E_{0}$, *and if* $R_{0}>1$, *then model* (4) *has a no*-*immune equilibrium* $E_{1}(x^{*}_{1},y^{*}_{1},v^{*}_{1},0)$, *except for equilibrium* $E_{0}$.
(iii) *If* $R_{0}>1$ *and* $R_{1}\leq1$, *then model* (4) *has only the virus*-*free equilibrium* $E_{0}$ *and the no*-*immune equilibrium* $E_{1}$, *and if* $R_{0}>1$ *and* $R_{1}>1$, *then model* (4) *has an infected equilibrium* $E_{2}(x^{*}_{2},y^{*}_{2},v^{*}_{2},z^{*}_{2})$, *except for equilibria* $E_{0}$ *and* $E_{1}$.

### Proof

It is clear that the equilibrium of model (4) satisfies the following equation:

11 $$ \textstyle\begin{cases} \lambda-d x-f(x,y,v)v=0,\\ f(x,y,v)v-ay-pyz=0,\\ ky-uv=0,\\ cyz-bz=0. \end{cases} $$

Obviously, (11) has a solution $(\frac{\lambda}{d},0,0,0)$. Hence, model (4) always has a virus-free equilibrium $E_{0}(\frac {\lambda}{d},0,0,0)$. This shows conclusion (i).

Let $z=0$, from (11) we have $y=\frac{\lambda-d x}{a}$, $v=\frac{k(\lambda-d x)}{au}$, and $f(x,y,v)v=ay$. Hence,

$$g_{1}(x)\triangleq f\biggl(x,\frac{\lambda-d x}{a},\frac{k(\lambda -d x)}{au} \biggr)-\frac{au}{k}=0. $$

We have $g_{1}(0)=-\frac{au}{k}<0$ and $g_{1}(\frac{\lambda}{d})=\frac {au}{k}(R_{0}-1)$. Based on $(A_{2})$ and $(A_{3})$, we know that $g_{1}(x)$ is monotonously increasing for all $x\in(0,\frac{\lambda}{d})$. When $R_{0}>1$, then $g_{1}(\frac{\lambda}{d})>0$. Hence, $g_{1}(x)=0$ has a unique solution $x_{1}^{*}\in(0,\frac{\lambda}{d})$. This shows that model (4) has a unique no-immune equilibrium $E_{1}(x^{*}_{1},y^{*}_{1},v^{*}_{1},0)$ with $y^{*}_{1}=\frac{\lambda -d x^{*}_{1}}{a}$ and $v^{*}_{1}=\frac{k(\lambda-d x^{*}_{1})}{au}$. When $R_{0}\leq1$, then $g_{1}(\frac{\lambda}{d})\leq0$. Hence, $g_{1}(x)=0$ has no solution in $(0,\frac{\lambda}{d})$. This shows that model (4) has only equilibrium $E_{0}$. Therefore, conclusion (ii) is true.

Let $z\neq0$, from (11) we have $y=\frac{b}{c}$, $v=\frac {kb}{uc}$, and the following equation:

$$g_{2}(x)\triangleq f\biggl(x,\frac{b}{c},\frac{kb}{uc} \biggr)-\frac {uc}{kb}(\lambda-d x)=0. $$

We have $g_{2}(0)=-\frac{uc\lambda}{kb}<0$. When $R_{0}>1$ and $R_{1}>1$, we know $y^{*}_{1}>\frac{b}{c}$. Owing to $y^{*}_{1}=\frac{\lambda-d x^{*}_{1}}{a}$, by simply calculating we can obtain $x^{*}_{1}<\frac{\lambda}{d }-\frac{ab}{d c}$. Hence,

$$ g_{2}\biggl(\frac{\lambda}{d}-\frac{ab}{d c}\biggr) =f\biggl( \frac{\lambda}{d}-\frac{ab}{d c},\frac{b}{c},\frac {kb}{uc} \biggr)-\frac{ua}{k} > f\bigl(x^{*}_{1},y^{*}_{1},v^{*}_{1} \bigr)-\frac{ua}{k}=0. $$

From $(A_{2})$, we know that $g_{2}(x)$ is monotonously increasing for $x>0$. Hence, there is a unique $x^{*}_{2}\in(0,\frac{\lambda }{d}-\frac{ab}{d c})$ such that $g_{2}(x^{*}_{2})=0$. This shows that model (4) has a unique infected equilibrium $E_{2}(x^{*}_{2},y^{*}_{2},v^{*}_{2},z^{*}_{2})$ with $y^{*}_{2}=\frac {b}{c}$, $v^{*}_{2}=\frac{kb}{uc}$ and

$$ z^{*}_{2} =\frac{1}{py^{*}_{2}}\bigl(\lambda-d x^{*}_{2}-ay^{*}_{2} \bigr) >\frac{1}{py^{*}_{2}} \biggl[\lambda-d \biggl(\frac{\lambda}{d }- \frac {ab}{d c}\biggr)-\frac{ab}{c} \biggr] =0. $$

When $R_{0}>1$ and $R_{1}\leq1$, similarly to above discussion we can see that if $g_{2}(x)=0$ has a positive solution $x_{2}^{*}$, then $x_{2}^{*}>\frac{\lambda}{d}-\frac{ab}{d c}$. But, if model (4) has a positive equilibrium $E_{2}(x^{*}_{2},y^{*}_{2},v^{*}_{2},z^{*}_{2})$, then $z^{*}_{2} =\frac{1}{py^{*}_{2}}(\lambda-d x^{*}_{2}-ay^{*}_{2})>0$. Hence, we must have $x_{2}^{*}<\frac{\lambda}{d}-\frac{ab}{d c}$, which leads to a contradiction. Therefore, conclusion (iii) is true. This completes the proof. □

## Stability of equilibria

First of all, we introduce the following assumption:

$$ \biggl(1-\frac{f(x,y,v)}{f(x,y^{*}_{i},v^{*}_{i})}\biggr) \biggl(\frac {f(x,y^{*}_{i},v^{*}_{i})}{f(x,y,v)}-\frac{v}{v^{*}_{i}} \biggr) \leq0, \quad i=1,2, \quad\quad\quad (A_{4})$$

for all $(x,y,v,z)\in\Gamma$, and $(x^{*}_{i},y^{*}_{i},v^{*}_{i})$ is the coordinate of equilibrium $E_{i}$ for $i=1,2 $, respectively.

Specially, when $f(x,y,v)=\frac{\beta x}{1+mx+nv}$, by simple calculation we know that $f(x,y,v)$ satisfies assumption $(A_{4})$.

However, when $f(x,y,v)=\frac{\beta x}{1+nv^{2}}$, in Section 4, we will give the numerical examples to indicate that assumption $(A_{4})$ may not be satisfied.

### Theorem 1

*Suppose that* $(A_{1})$-$(A_{3})$ *hold*. *If* $R_{0}\leq1$, *then the virus*-*free equilibrium* $E_{0}(\frac{\lambda}{d},0,0,0)$ *of model* (4) *is globally asymptotically stable*.

### Proof

Let $x^{*}=\frac{\lambda}{d}$ and $(x_{n},y_{n},v_{n},z_{n})$ be any solution of model (3) with initial condition (6). Choosing a Lyapunov function as follows:

$$W_{n}=x_{n}-x^{*}- \int^{x_{n}}_{x^{*}}\frac {f(x^{*},0,0)}{f(s,0,0)}\,\mathrm {d}s+y_{n}+\frac{a(1+\phi u)}{k}v_{n}+\frac {p}{c}z_{n}, $$

we let

$$m(x)\triangleq x-x^{*}- \int^{x}_{x^{*}}\frac {f(x^{*},0,0)}{f(s,0,0)}\,\mathrm {d}s. $$

According to $(A_{2})$, we easily obtain $m(x)\geq m(x^{*})=0$ for all $x\geq0$. Therefore, $W_{n}\geq0$ for all $x_{n}\geq0$, $y_{n}\geq0$, $v_{n}\geq0$, and $z_{n}\geq0$. In addition, $W_{n}=0$ if and only if $x_{n}=x^{*}$, $y_{n}=0$, $v_{n}=0$ and $z_{n}=0$. Computing $\Delta W_{n}$, we have

$$\begin{aligned} \Delta W_{n} = & x_{n+1}-x_{n}- \int^{x_{n+1}}_{x_{n}}\frac {f(x^{*},0,0)}{f(s,0,0)}\,\mathrm {d}s+y_{n+1} -y_{n} \\ &{} +\frac{a(1+\phi u)}{k}(v_{n+1}-v_{n})+ \frac {p}{c}(z_{n+1}-z_{n}) \\ \leq & \biggl(1-\frac{f(x^{*},0,0)}{f(x_{n+1},0,0)} \biggr) (x_{n+1}-x_{n})+y_{n+1} -y_{n} \\ &{} +\frac{a(1+\phi u)}{k}(v_{n+1}-v_{n})+ \frac {p}{c}(z_{n+1}-z_{n}). \end{aligned}$$

Substituting model (4), we have

$$\begin{aligned} \Delta W_{n} \leq & \phi \biggl[ \biggl(1-\frac {f(x^{*},0,0)}{f(x_{n+1},0,0)} \biggr) \bigl(\lambda -d x_{n+1}-f(x_{n+1},y_{n},v_{n})v_{n} \bigr) +f(x_{n+1},y_{n},v_{n})v_{n} \\ & {}-ay_{n+1}-py_{n+1}z_{n+1}+\frac{a(1+\phi u)}{k}(ky_{n+1}-uv_{n+1})+ \frac{p}{c}(cy_{n+1}z_{n+1}-bz_{n+1}) \biggr] \\ = & \phi \biggl[ \biggl(1-\frac {f(x^{*},0,0)}{f(x_{n+1},0,0)} \biggr) (\lambda -d x_{n+1})-f(x_{n+1},y_{n},v_{n})v_{n} \\ & {} +\frac {f(x_{n+1},y_{n},v_{n})}{f(x_{n+1},0,0)}f\bigl(x^{*},0,0\bigr)v_{n}+f(x_{n+1},y_{n},v_{n})v_{n} -ay_{n+1}-py_{n+1}z_{n+1} \\ & {}+ay_{n+1}+\phi auy_{n+1}-\frac{au(1+\phi u)}{k}v_{n+1}+py_{n+1}z_{n+1} -\frac{pb}{c}z_{n+1} \biggr]. \end{aligned}$$

Substituting $\lambda=d x^{*}$ and $v_{n+1}=\frac{v_{n}+\phi ky_{n+1}}{1+\phi u}$ from model (7), we further obtain

$$\begin{aligned} \Delta W_{n} \leq& \phi \biggl[d x^{*} \biggl(1- \frac {x_{n+1}}{x^{*}} \biggr) \biggl(1-\frac{f(x^{*},0,0)}{f(x_{n+1},0,0)} \biggr) + \frac{f(x_{n+1},y_{n},v_{n})}{f(x_{n+1},0,0)}f\bigl(x^{*},0,0\bigr)v_{n} \\ &{} +\phi auy_{n+1} -\frac{au(1+\phi u)}{k}\frac{v_{n}+\phi ky_{n+1}}{1+\phi u}- \frac {pb}{c}z_{n+1} \biggr] \\ =& \phi \biggl[d x^{*} \biggl(1-\frac{x_{n+1}}{x^{*}} \biggr) \biggl(1- \frac{f(x^{*},0,0)}{f(x_{n+1},0,0)} \biggr) \\ &{}+\frac{au}{k}\biggl(\frac {f(x_{n+1},y_{n},v_{n})}{f(x_{n+1},0,0)}R_{0}-1 \biggr)v_{n}-\frac {pb}{c}z_{n+1} \biggr]. \end{aligned}$$

Based on $(A_{3})$, we have

$$\Delta W_{n}\leq\phi \biggl[d x^{*} \biggl(1- \frac{x_{n+1}}{x^{*}} \biggr) \biggl(1-\frac{f(x^{*},0,0)}{f(x_{n+1},0,0)} \biggr) + \frac{au}{k}(R_{0}-1)v_{n}-\frac{pb}{c}z_{n+1} \biggr]. $$

According to $(A_{2})$, we know

$$\biggl(1-\frac{x_{n+1}}{x^{*}} \biggr) \biggl(1-\frac {f(x^{*},0,0)}{f(x_{n+1},0,0)} \biggr)\leq0. $$

Therefore, when $R_{0}\leq1$ and $z_{n}>0$, $v_{n}>0$, we get $\Delta W_{n}\leq0$. It is obvious that $\Delta W_{n}=0$ if and only if $x_{n}=x^{*}$, $y_{n}=0$, $v_{n}=0$ and $z_{n}=0$. Based on LaSalle’s invariance principle (see [33]), we finally see that the virus-free equilibrium $E_{0}(x^{*},0,0,0)$ is globally asymptotically stable. This completes the proof. □

### Theorem 2

*Suppose that* $(A_{1})$-$(A_{3})$ *hold*. *If* $R_{0}>1$, *then the virus*-*free equilibrium* $E_{0}(\frac{\lambda}{d},0,0,0)$ *of model* (4) *is unstable*.

### Proof

By calculating, we can see that the linearization system of model (4) at equilibrium $E_{0}$ is

12 $$ \textstyle\begin{cases} X_{n+1} =\frac{1}{1+\phi d} X_{n}-\frac{\phi f(\frac {\lambda}{d},0,0)}{1+\phi d}V_{n},\\ Y_{n+1} =\frac{1}{1+\phi a}Y_{n}+\frac{\phi f(\frac {\lambda}{d},0,0)}{1+\phi a}V_{n},\\ V_{n+1} =\frac{\phi k}{(1+\phi a)(1+\phi u)}Y_{n}+\frac {1+\phi[a+f(\frac{\lambda}{d},0,0)]}{(1+\phi a)(1+\phi u)}V_{n},\\ Z_{n+1} =\frac{1}{1+\phi b}Z_{n}. \end{cases} $$

By calculating we can obtain the characteristic equation of system (12),

$$\begin{aligned} f(\lambda) \triangleq& \biggl(\lambda-\frac{1}{1+\phi d} \biggr) \biggl(\lambda- \frac{1}{1+\phi b} \biggr) \biggl[(1+\phi a) (1+\phi u)\lambda^{2} \\ &{} -\biggl(2+\phi\biggl[a+u+\phi kf\biggl(\frac{\lambda}{d},0,0\biggr)\biggr] \biggr)\lambda +1 \biggr]=0. \end{aligned}$$

Solving this equation, we get $\lambda_{1}=\frac{1}{1+\phi d}$, $\lambda_{2}=\frac{1}{1+\phi b}$, $\lambda_{3}$ and $\lambda_{4}$ are determined by the following equation:

$$g(\lambda)\triangleq(1+\phi a) (1+\phi u)\lambda^{2}-\biggl(2+\phi \biggl[a+u+\phi kf\biggl(\frac{\lambda}{d},0,0\biggr)\biggr]\biggr)\lambda+1=0. $$

Since when $R_{0}>1$, we have $g(1)=\phi^{2}au(1-R_{0})<0$ and $\lim_{\lambda\to\infty}g(\lambda)=\infty$, there exists an $\eta\in(1,+\infty)$ such that $g(\eta)=0$. This shows that $\lambda_{3}$ or $\lambda_{4}$ is greater than 1. Therefore, the virus-free equilibrium $E_{0}(\frac{\lambda }{d},0,0,0)$ is unstable. This completes the proof. □

For model (5), by calculating, we see that the basic reproductive numbers $R_{0}$ and $R_{1}$ are given by

$$R_{0}=\frac{k\beta\lambda}{au(d+m\lambda)},\quad \quad R_{1}=\frac{\lambda \beta kc+a^{2}umb}{aduc+adbkn+a\beta bk+au\lambda mc}. $$

As a consequence of Theorem 1 and Theorem 2 we have the following result for model (5).

### Corollary 1

*If* $R_{0}\leq1$, *then the virus*-*free equilibrium* $E_{0}(\frac{\lambda }{d},0,0,0)$ *of model* (5) *is globally asymptotically stable*. *Otherwise*, *if* $R_{0}>1$, *then equilibrium* $E_{0}$ *is unstable*.

### Theorem 3

*Suppose that* $(A_{1})$-$(A_{3})$ *and* $(A_{4})$ *for* $i=1$ *hold*. *If* $R_{0}>1$ *and* $R_{1}\leq1$, *then the no*-*immune equilibrium* $E_{1}(x^{*}_{1},y^{*}_{1},v^{*}_{1},0)$ *of model* (4) *is globally asymptotically stable*.

### Proof

Let $(x_{n},y_{n},v_{n},z_{n})$ be any solution of model (4) with initial condition (6). From Lemma 2, we can assume $(x_{n},y_{n},v_{n},z_{n})\in\Gamma$ for all $n\geq0$. Define a Lyapunov function as follows:

$$\begin{aligned} L_{n} =& x_{n}-x^{*}_{1}- \int^{x_{n}}_{x^{*}_{1}} \frac{f(x^{*}_{1},y^{*}_{1},v^{*}_{1})}{f(s,y^{*}_{1},v^{*}_{1})}\,\mathrm {d}s +y_{n}-y^{*}_{1}-y^{*}_{1}\ln \frac{y_{n}}{y^{*}_{1}} \\ &{} +\frac{a(1+\phi u)}{k} \biggl(v_{n}-v^{*}_{1}-v^{*}_{1} \ln\frac{v_{n}}{v^{*}_{1}} \biggr)+\frac {p}{c}z_{n+1}. \end{aligned}$$

According to $(A_{2})$, we easily obtain

$$m(x)\triangleq x-x^{*}_{1}- \int^{x}_{x^{*}_{1}}\frac {f(x^{*}_{1},0,0)}{f(s,0,0)}\,\mathrm {d}s>0 $$

for all $x\geq0$ and $x\neq x^{*}$. Hence, $L_{n}\geq0$ for all $x_{n}\geq0$, $y_{n}\geq0$, $v_{n}\geq0$ and $z_{n}\geq0$. Obviously, $L_{n}=0$ if and only if $x_{n}=x^{*}_{1}$, $y_{n}=y^{*}_{1}$, $v_{n}=v^{*}_{1}$, and $z_{n}=0$. Computing $\Delta L_{n}$, we have

$$\begin{aligned} \Delta L_{n} =& x_{n+1}-x_{n}- \int^{x_{n+1}}_{x_{n}} \frac{f(x^{*}_{1},y^{*}_{1},v^{*}_{1})}{f(s,y^{*}_{1},v^{*}_{1})}\,\mathrm {d}s+y_{n+1} -y_{n}-y^{*}_{1}\ln\frac{y_{n+1}}{y_{n}} \\ &{} +\frac{a(1+\phi u)}{k}(v_{n+1}-v_{n})-\frac{a(1+\phi u)}{k}v^{*}_{1} \ln\frac{v_{n+1}}{v_{n}}+\frac{p}{c}(z_{n+1}-z_{n}) \\ \leq& \biggl(1-\frac {f(x^{*}_{1},y^{*}_{1},v^{*}_{1})}{f(x_{n+1},y^{*}_{1},v^{*}_{1})} \biggr) (x_{n+1}-x_{n}) +y_{n+1}-y_{n}-y^{*}_{1}\ln \frac{y_{n+1}}{y_{n}} \\ &{} -\frac{a(1+\phi u)}{k}v^{*}_{1}\ln\frac {v_{n+1}}{v_{n}}+ \frac{p}{c}(z_{n+1}-z_{n})+\frac{a(1+\phi u)}{k}(v_{n+1}-v_{n}). \end{aligned}$$

From $\ln x\leq x-1$ for $x>0$, we further have

$$\begin{aligned} \Delta L_{n} \leq& \biggl(1-\frac {f(x^{*}_{1},y^{*}_{1},v^{*}_{1})}{f(x_{n+1},y^{*}_{1},v^{*}_{1})} \biggr) (x_{n+1}-x_{n}) + \biggl(1-\frac{y^{*}_{1}}{y_{n+1}} \biggr) (y_{n+1}-y_{n}) \\ &{} +\frac{a}{k} \biggl(1-\frac{v^{*}_{1}}{v_{n+1}} \biggr) (v_{n+1}-v_{n})+\frac{\phi au}{k} \biggl(v_{n+1}-v_{n}+v^{*}_{1} \ln \frac{v_{n}}{v_{n+1}} \biggr) +\frac{p}{c}(z_{n+1}-z_{n}). \end{aligned}$$

Substituting model (4), owing to $x^{*}_{1}$, $y^{*}_{1}$, and $v^{*}_{1}$ satisfying the equations

$$ \textstyle\begin{cases} 0= \lambda-d x^{*}_{1}-f(x^{*}_{1},y^{*}_{1},v^{*}_{1})v^{*}_{1},\\ 0= f(x^{*}_{1},y^{*}_{1},v^{*}_{1})v^{*}_{1}-ay^{*}_{1},\\ 0= ky^{*}_{1}-uv^{*}_{1}, \end{cases} $$

we obtain

$$\begin{aligned} \Delta L_{n} \leq& \biggl(1-\frac {f(x^{*}_{1},y^{*}_{1},v^{*}_{1})}{f(x_{n+1},y^{*}_{1},v^{*}_{1})} \biggr) (x_{n+1}-x_{n}) + \biggl(1-\frac{y^{*}_{1}}{y_{n+1}} \biggr) \bigl(f(x_{n+1},y_{n},v_{n})v_{n} \\ &{} -ay_{n+1}-py_{n+1}z_{n+1}\bigr)\phi+ \frac{a}{k} \biggl(1-\frac{v^{*}_{1}}{v_{n+1}} \biggr) (ky_{n+1}-uv_{n+1}) \phi \\ & +\frac{\phi au}{k}(v_{n+1}-v_{n}) +\frac{\phi au}{k}v^{*}_{1} \ln\frac{v_{n}}{v_{n+1}}+\frac {p}{c}(cy_{n+1}z_{n+1}-bz_{n+1}) \phi \\ =& \biggl(1-\frac {f(x^{*}_{1},y^{*}_{1},v^{*}_{1})}{f(x_{n+1},y^{*}_{1},v^{*}_{1})} \biggr) (x_{n+1}-x_{n}) +ay^{*}_{1} \biggl(1-\frac{y^{*}_{1}}{y_{n+1}}\frac{v_{n}}{v^{*}_{1}} \frac{f(x_{n+1},y_{n},v_{n})}{f(x^{*}_{1},y^{*}_{1},v^{*}_{1})} \biggr)\phi \\ &{} +ay^{*}_{1} \biggl(1-\frac{y_{n+1}}{v_{n+1}} \frac{v^{*}_{1}}{y^{*}_{1}}-\frac{v_{n}}{v^{*}_{1}} \biggr)\phi +f(x_{n+1},y_{n},v_{n})v_{n} \phi \\ &{} +\frac{\phi pb}{c}(R_{1}-1)z_{n+1}+ \frac{\phi au}{k}v^{*}_{1} \ln\frac{v_{n}}{v_{n+1}}. \end{aligned}$$

Since $\lambda=d x^{*}_{1}+ay^{*}_{1}$, the first equation model (4) becomes

$$x_{n+1}=x_{n}+\phi\bigl[d x^{*}_{1}+ay^{*}_{1}-d x_{n+1}-f(x_{n+1},y_{n},v_{n})v_{n} \bigr]. $$

We have

$$\begin{aligned} \Delta L_{n} \leq& \biggl(1-\frac {f(x^{*}_{1},y^{*}_{1},v^{*}_{1})}{f(x_{n+1},y^{*}_{1},v^{*}_{1})} \biggr) \bigl(d x^{*}_{1} +ay^{*}_{1}-d x_{n+1}-f(x_{n+1},y_{n},v_{n})v_{n} \bigr)\phi \\ &{} +ay^{*}_{1} \biggl(1-\frac{y^{*}_{1}}{y_{n+1}} \frac {v_{n}}{v^{*}_{1}} \frac{f(x_{n+1},y_{n},v_{n})}{f(x^{*}_{1},y^{*}_{1},v^{*}_{1})} \biggr)\phi+ay^{*}_{1} \biggl(1-\frac{y_{n+1}}{v_{n+1}} \frac{v^{*}_{1}}{y^{*}_{1}}-\frac{v_{n}}{v^{*}_{1}} \biggr)\phi \\ &{} +\phi f(x_{n+1},y_{n},v_{n})v_{n}+ \frac{\phi pb}{c}(R_{1}-1)z_{n+1}+\frac{\phi au}{k}v^{*}_{1} \ln\frac{v_{n}}{v_{n+1}} \\ =& \biggl(1-\frac {f(x^{*}_{1},y^{*}_{1},v^{*}_{1})}{f(x_{n+1},y^{*}_{1},v^{*}_{1})} \biggr) \bigl(d x^{*}_{1}-d x_{n+1} \bigr)\phi+ay^{*}_{1} \biggl(1- \frac {f(x^{*}_{1},y^{*}_{1},v^{*}_{1})}{f(x_{n+1},y^{*}_{1},v^{*}_{1})} \\ &{}+\frac {f(x_{n+1},y_{n},v_{n})}{f(x_{n+1},y^{*}_{1},v^{*}_{1})}\frac {v_{n}}{v^{*}_{1}} \biggr)\phi+ay^{*}_{1} \biggl(1-\frac {y^{*}_{1}}{y_{n+1}}\frac{v_{n}}{v^{*}_{1}} \frac{f(x_{n+1},y_{n},v_{n})}{f(x^{*}_{1},y^{*}_{1},v^{*}_{1})} \biggr)\phi \\ &{}+ay^{*}_{1}\biggl(1-\frac{y_{n+1}}{v_{n+1}} \frac {v^{*}_{1}}{y^{*}_{1}} -\frac{v_{n}}{v^{*}_{1}}\biggr)\phi+\frac{\phi pb}{c}(R_{1}-1)z_{n+1} +\frac{\phi au}{k}v^{*}_{1}\ln\frac{v_{n}}{v_{n+1}} \\ =& \phi d x^{*}_{1} \biggl(1-\frac{x_{n+1}}{x^{*}_{1}} \biggr) \biggl(1-\frac {f(x^{*}_{1},y^{*}_{1},v^{*}_{1})}{f(x_{n+1},y^{*}_{1},v^{*}_{1})} \biggr) +\phi ay^{*}_{1} \biggl(4-\frac {f(x^{*}_{1},y^{*}_{1},v^{*}_{1})}{f(x_{n+1},y^{*}_{1},v^{*}_{1})} \\ &{} -\frac{y^{*}_{1}}{y_{n+1}}\frac{v_{n}}{v^{*}_{1}} \frac{f(x_{n+1},y_{n},v_{n})}{f(x^{*}_{1},y^{*}_{1},v^{*}_{1})} - \frac{y_{n+1}}{y^{*}_{1}}\frac{v^{*}_{1}}{v_{n+1}} -\frac{f(x_{n+1},y^{*}_{1},v^{*}_{1})}{f(x_{n+1},y_{n},v_{n})}-\ln \frac{v_{n+1}}{v_{n}} \biggr) \\ &{} +\phi ay^{*}_{1} \biggl(-1-\frac{v_{n}}{v^{*}_{1}} + \frac{f(x_{n+1},y^{*}_{1},v^{*}_{1})}{f(x_{n+1},y_{n},v_{n})}+\frac {v_{n}}{v^{*}_{1}} \frac{f(x_{n+1},y_{n},v_{n})}{f(x_{n+1},y^{*}_{1},v^{*}_{1})} \biggr) +\frac{\phi pb}{c}(R_{1}-1)z_{n+1}. \end{aligned}$$

Let $g(x)=x-1-\ln x$, then $g(x)\geq0$ for all $x>0$. Hence, we can get

13 $$\begin{aligned}& 4-\frac {f(x^{*}_{1},y^{*}_{1},v^{*}_{1})}{f(x_{n+1},y^{*}_{1},v^{*}_{1})} -\frac{y^{*}_{1}}{y_{n+1}}\frac{v_{n}}{v^{*}_{1}} \frac{f(x_{n+1},y_{n},v_{n})}{f(x^{*}_{1},y^{*}_{1},v^{*}_{1})} \\& \quad\quad{} -\frac{y_{n+1}}{y^{*}_{1}}\frac{v^{*}_{1}}{v_{n+1}} -\frac{f(x_{n+1},y^{*}_{1},v^{*}_{1})}{f(x_{n+1},y_{n},v_{n})}-\ln \frac{v_{n+1}}{v_{n}} \\& \quad = -g\biggl(\frac {f(x^{*}_{1},y^{*}_{1},v^{*}_{1})}{f(x_{n+1},y^{*}_{1},v^{*}_{1})}\biggr) -g\biggl(\frac{y^{*}_{1}}{y_{n+1}} \frac{v_{n}}{v^{*}_{1}} \frac{f(x_{n+1},y_{n},v_{n})}{f(x^{*}_{1},y^{*}_{1},v^{*}_{1})}\biggr) \\& \quad\quad{} -g\biggl(\frac{y_{n+1}}{y^{*}_{1}}\frac{v^{*}_{1}}{v_{n+1}}\biggr) -g\biggl( \frac{f(x_{n+1},y^{*}_{1},v^{*}_{1})}{f(x_{n+1},y_{n},v_{n})}\biggr) \leq0. \end{aligned}$$

According to $(A_{2})$, we know

14 $$ \biggl(1-\frac{x_{n+1}}{x^{*}_{1}} \biggr) \biggl(1-\frac {f(x^{*}_{1},y^{*}_{1},v^{*}_{1})}{f(x_{n+1},y^{*}_{1},v^{*}_{1})} \biggr) \leq0. $$

Since $(A_{4})$ holds for $i=1$, we further have

15 $$ -1-\frac{v_{n}}{v^{*}_{1}} + \frac{f(x_{n+1},y^{*}_{1},v^{*}_{1})}{f(x_{n+1},y_{n},v_{n})}+\frac {v_{n}}{v^{*}_{1}} \frac{f(x_{n+1},y_{n},v_{n})}{f(x_{n+1},y^{*}_{1},v^{*}_{1})}\leq0. $$

Therefore, when $R_{1}\leq1$, from (13), (14), and (15) we finally obtain $\Delta L_{n}\leq0$ and $\Delta L_{n}=0$ if and only if $x_{n}=x^{*}_{1}$, $y_{n}=y^{*}_{1}$, $v_{n}=v^{*}_{1}$ and $z_{n}=0$. Based on LaSalle’s invariance principle, we see that the no-immune equilibrium $E_{1}(x^{*}_{1}, y^{*}_{1}, v^{*}_{1},0)$ is globally asymptotically stable. This completes the proof. □

### Theorem 4

*Suppose that* $(A_{1})$-$(A_{3})$ *hold*. *If* $R_{0}>1$ *and* $R_{1}>1$, *then the no*-*immune equilibrium* $E_{1}(x^{*}_{1},y^{*}_{1},v^{*}_{1},0)$ *of model* (4) *is unstable*.

### Proof

By calculating, we easily see that the linearization system of model (4) at equilibrium $E_{1}(x^{*}_{1},y^{*}_{1},v^{*}_{1},0)$ is

16 $$ \textstyle\begin{cases} X_{n+1}= \frac{1}{1+\phi(d+\frac{\partial f}{\partial x}v^{*}_{1})}X_{n}-\frac{\phi\frac{\partial f}{\partial y}v^{*}_{1}}{1+\phi(d+\frac{\partial f}{\partial x}v^{*}_{1})}Y_{n}-\frac{\phi(\frac{\partial f}{\partial v}v^{*}_{1}+f(x^{*}_{1},y^{*}_{1},v^{*}_{1}))}{1+\phi(d+\frac {\partial f}{\partial x}v^{*}_{1})}V_{n},\\ Y_{n+1}= \frac{\phi\frac{\partial f}{\partial x}v^{*}_{1}}{(1+\phi (d+\frac{\partial f}{\partial x}v^{*}_{1}))(1+\phi a)}X_{n}+(1-\phi^{2} \frac{\frac{\partial f}{\partial x}v^{*}_{1}\frac{\partial f}{\partial y}v^{*}_{1}}{1+\phi(d+\frac{\partial f}{\partial x}v^{*}_{1})}+\phi\frac{\partial f}{\partial y}v^{*}_{1}) \\ \hphantom{Y_{n+1}=}{} \times\frac{1}{1+\phi a}Y_{n}+\frac{1}{1+\phi a}[-\phi\frac {\partial f}{\partial x}v^{*}_{1}\frac{\phi(\frac{\partial f}{\partial v}v^{*}_{1}+f(x^{*}_{1},y^{*}_{1},v^{*}_{1}))}{1+\phi (d+\frac{\partial f}{\partial x}v^{*}_{1})}\\ \hphantom{Y_{n+1}=}{}+\phi\frac{\partial f}{\partial v}v^{*}_{1}+\phi f(x^{*}_{1},y^{*}_{1},v^{*}_{1})]V_{n}-\frac{\phi py^{*}_{1}}{1+\phi (b-cy^{*}_{1})}\frac{1}{1+\phi a}Z_{n},\\ V_{n+1}= \frac{\phi\frac{\partial f}{\partial x}v^{*}_{1}}{(1+\phi (d+\frac{\partial f}{\partial x}v^{*}_{1}))(1+\phi a)}\frac{\phi k}{1+\phi u}X_{n}+(1-\phi^{2} \frac{\frac{\partial f}{\partial x}v^{*}_{1}\frac{\partial f}{\partial y}v^{*}_{1}}{1+\phi(d+\frac {\partial f}{\partial x}v^{*}_{1})}\\ \hphantom{V_{n+1}=}{} +\phi\frac{\partial f}{\partial y}v^{*}_{1})\frac{1}{1+\phi a}\frac {\phi k}{1+\phi u}Y_{n}+[\frac{1}{1+\phi a}\frac{\phi k}{1+\phi u}(-\phi\frac{\partial f}{\partial x}v^{*}_{1}\\ \hphantom{V_{n+1}=}{}\times\frac{\phi(\frac{\partial f}{\partial v}v^{*}_{1}+f(x^{*}_{1},y^{*}_{1},v^{*}_{1}))}{1+\phi(d+\frac {\partial f}{\partial x}v^{*}_{1})}+\phi\frac{\partial f}{\partial v}v^{*}_{1}+\phi f(x^{*}_{1},y^{*}_{1},v^{*}_{1}))+\frac{1}{1+\phi u}]V_{n}\\ \hphantom{V_{n+1}=}{}-\frac{\phi py^{*}_{1}}{1+\phi(b-cy^{*}_{1})}\frac{1}{1+\phi a}\frac{\phi k}{1+\phi u}Z_{n},\\ Z_{n+1}= \frac{1}{1+\phi(b-cy^{*}_{1})}Z_{n}, \end{cases} $$

where

$$\frac{\partial f}{\partial x}=\frac{\partial f}{\partial x}\bigl(x^{*}_{1},y^{*}_{1},v^{*}_{1} \bigr), \quad \quad\frac{\partial f}{\partial y}=\frac{\partial f}{\partial y}\bigl(x^{*}_{1},y^{*}_{1},v^{*}_{1} \bigr),\quad \quad \frac{\partial f}{\partial v}=\frac{\partial f}{\partial v}\bigl(x^{*}_{1},y^{*}_{1},v^{*}_{1} \bigr). $$

By calculating we obtain the characteristic equation of equation (16),

$$f(\lambda)\triangleq \biggl(\lambda-\frac{1}{1+\phi(b-cy^{*}_{1})} \biggr) \bigl(\lambda ^{3}+m\lambda^{2}+n\lambda+l\bigr)=0, $$

where

$$\begin{aligned}& m= -(1+\phi d)\frac{\phi^{2} k(\frac{\partial f}{\partial v}v^{*}_{1}+f(x^{*}_{1},y^{*}_{1},v^{*}_{1}))}{(1+\phi (d+\frac{\partial f}{\partial x}v^{*}_{1}))(1+\phi u)(1+\phi a)}-\frac {1}{1+\phi u} \\& \hphantom{m=}{}-\frac{\phi\frac{\partial f}{\partial y}v^{*}_{1}+1}{1+\phi a}+\frac{\phi^{2} \frac{\partial f}{\partial x}v^{*}_{1}\frac{\partial f}{\partial y}v^{*}_{1}}{(1+\phi(d+\frac {\partial f}{\partial x}v^{*}_{1}))(1+\phi a)}-\frac{1}{1+\phi (d+\frac{\partial f}{\partial x}v^{*}_{1})}, \\& n= \frac{\phi^{2} k(\frac{\partial f}{\partial v}v^{*}_{1}+f(x^{*}_{1},y^{*}_{1},v^{*}_{1}))}{(1+\phi(d+\frac {\partial f}{\partial x}v^{*}_{1}))(1+\phi u)(1+\phi a)}+\frac {1}{(1+\phi(d+\frac{\partial f}{\partial x}v^{*}_{1}))(1+\phi u)} \\& \hphantom{n=}{} + \biggl(\frac{1}{1+\phi(d+\frac{\partial f}{\partial x}v^{*}_{1})}+\frac{1}{1+\phi u} \biggr) \frac{\phi\frac{\partial f}{\partial y}v^{*}_{1}+1}{1+\phi a} \\& \hphantom{n=}{} -\frac{\phi^{2} k\frac{\partial f}{\partial x}v^{*}_{1}}{(1+\phi(d+\frac{\partial f}{\partial x}v^{*}_{1}))(1+\phi u)^{2}(1+\phi a)}, \\& l= -\frac{\phi\frac{\partial f}{\partial y}v^{*}_{1}+1}{(1+\phi(d+m))(1+\phi u)(1+\phi a)}. \end{aligned}$$

Let $\lambda_{i}$ ($i=1,2,3,4$) be the roots of $f(\lambda)=0$, then $\lambda_{1}=\frac{1}{1+\phi(b-cy^{*}_{1})}$ and $\lambda_{2}$, $\lambda_{3}$ and $\lambda_{4}$ satisfy the equation $\lambda^{3}+m\lambda^{2}+n\lambda+l=0$. From $Z_{n}>0$, we know $\frac{1}{1+\phi(b-cy^{*}_{1})}>0$. By $R_{1}>1$, we have $\frac{b}{c}< y^{*}_{1}$. Hence, we get $\frac {1}{1+\phi(b-cy^{*}_{1})}>1$. This shows that when $R_{1}>1$, the no-immune equilibrium $E_{1}(x^{*}_{1},y^{*}_{1},v^{*}_{1},0)$ is unstable. This completes the proof. □

As a consequence of Theorems 3 and 4 we have the following result for model (5).

### Corollary 2

*If* $R_{0}>1$ *and* $R_{1}\leq1$, *then the no*-*immune equilibrium* $E_{1}(x^{*}_{1},y^{*}_{1},v^{*}_{1},0)$ *of model* (5) *is globally asymptotically stable*. *Otherwise*, *if* $R_{0}>1$ *and* $R_{1}>1$, *then equilibrium* $E_{1}$ *is unstable*.

### Theorem 5

*Suppose that* $(A_{1})$-$(A_{3})$ *and* $(A_{4})$ *for* $i=2$ *hold*. *If* $R_{0}>1$ *and* $R_{1}>1$, *then the infected equilibrium* $E_{2}(x^{*}_{2},y^{*}_{2},v^{*}_{2},z^{*}_{2})$ *of model* (4) *is globally asymptotically stable*.

### Proof

Let $(x_{n},y_{n},v_{n},z_{n})$ be any solution of model (4) with initial condition (6). We can assume by Lemma 2 $(x_{n},y_{n},v_{n},z_{n})\in\Gamma$ for all $n\geq0$. Define a Lyapunov function as follows:

$$\begin{aligned} L_{n} =& x_{n}-x^{*}_{2}- \int^{x_{n}}_{x^{*}_{2}} \frac {f(x^{*}_{2},y^{*}_{2},v^{*}_{2})}{f(s,y^{*}_{2},v^{*}_{2})}\,\mathrm {d}s +y_{n}-y^{*}_{2}-y^{*}_{2} \ln\frac{y_{n}}{y^{*}_{2}} \\ &{} + \frac{(a+pz^{*}_{2})(1+\phi u)}{k} \biggl(v_{n}-v^{*}_{2}-v^{*}_{2} \ln \frac{v_{n}}{v^{*}_{2}} \biggr) +\frac{p}{c} \biggl(z_{n}-z^{*}_{2}-z^{*}_{2} \ln\frac {z_{n}}{z^{*}_{2}} \biggr). \end{aligned}$$

Obviously, we know $L_{n}\geq0$ for all $x_{n}\geq0$, $y_{n}\geq0$, $v_{n}\geq0$ and $z_{n}\geq0$, and $L_{n}=0$ if and only if $x_{n}=x^{*}_{2}$, $y_{n}=y^{*}_{2}$, $v_{n}=v^{*}_{2}$, and $z_{n}=z^{*}_{2}$. Computing $\Delta L_{n}$, we have

$$\begin{aligned} \Delta L_{n} =& x_{n+1}-x_{n}- \int^{x_{n+1}}_{x_{n}}\frac {f(x^{*}_{2},y^{*}_{2},v^{*}_{2})}{f(s,y^{*}_{2},v^{*}_{2})}\,\mathrm {d}s +y_{n+1}-y_{n}-y^{*}_{2}\ln \frac{y_{n+1}}{y_{n}} \\ &{} +\frac{(a+pz^{*}_{2})(1+\phi u)}{k} \biggl(v_{n+1}-v_{n} -v^{*}_{2}\ln\frac{v_{n+1}}{v_{n}} \biggr)+\frac{p}{c} \biggl(z_{n+1}-z_{n}-z^{*}_{2}\ln \frac{z_{n+1}}{z_{n}} \biggr) \\ \leq& \biggl(1-\frac {f(x^{*}_{2},y^{*}_{2},v^{*}_{2})}{f(x_{n+1},y^{*}_{2},v^{*}_{2})} \biggr) (x_{n+1}-x_{n}) +y_{n+1}-y_{n}+y^{*}_{2}\ln \frac{y_{n}}{y_{n+1}} \\ &{}+\frac{(a+pz^{*}_{2})(1+\phi u)}{k} \biggl(v_{n+1}-v_{n} -v^{*}_{2}\ln\frac{v_{n+1}}{v_{n}} \biggr)+\frac{p}{c} \biggl(z_{n+1}-z_{n}-z^{*}_{2}\ln \frac{z_{n+1}}{z_{n}} \biggr). \end{aligned}$$

Using $\ln x\leq x-1$ for $x>0$, we further have

$$\begin{aligned} \Delta L_{n} \leq& \biggl(1-\frac {f(x^{*}_{2},y^{*}_{2},v^{*}_{2})}{f(x_{n+1},y^{*}_{2},v^{*}_{2})} \biggr) (x_{n+1}-x_{n}) + \biggl(1-\frac{y^{*}_{2}}{y_{n+1}} \biggr) (y_{n+1}-y_{n}) \\ &{} +\frac{(a+pz^{*}_{2})(1+\phi u)}{k}(v_{n+1}-v_{n})- \frac{(a+pz^{*}_{2})(1+\phi u)}{k}v^{*}_{2}\ln \frac{v_{n+1}}{v_{n}} \\ &{} +\frac{p}{c} \biggl(1 -\frac{z^{*}_{2}}{z_{n+1}} \biggr) (z_{n+1}-z_{n}). \end{aligned}$$

Since equilibrium $E_{2}(x_{2}^{*},y_{2}^{*},v_{2}^{*},z_{2}^{*})$ satisfies the equations

$$\textstyle\begin{cases} \lambda-d x_{2}^{*}-f(x_{2}^{*},y_{2}^{*},v_{2}^{*})v_{2}^{*}=0,\\ f(x_{2}^{*},y_{2}^{*},v_{2}^{*})v_{2}^{*}-ay_{2}^{*}-py_{2}^{*}z_{2}^{*}=0,\\ ky_{2}^{*}-uv_{2}^{*}=0,\\ cy_{2}^{*}z_{2}^{*}-bz_{2}^{*}=0, \end{cases} $$

we have

$$\begin{aligned} \Delta L_{n} \leq& \biggl(1-\frac {f(x^{*}_{2},y^{*}_{2},v^{*}_{2})}{f(x_{n+1},y^{*}_{2},v^{*}_{2})} \biggr) (x_{n+1}-x_{n})+ \biggl(1-\frac{y^{*}_{2}}{y_{n+1}} \biggr) (y_{n+1}-y_{n}) \\ &{} +\frac{(a+pz^{*}_{2})(1+\phi u)}{k}(v_{n+1}-v_{n})- \frac{(a+pz^{*}_{2})}{k}v^{*}_{2}\ln\frac {v_{n+1}}{v_{n}} \\ &{} -\phi\bigl(ay^{*}_{2}+py^{*}_{2}z^{*}_{2} \bigr) \ln\frac{v_{n+1}}{v_{n}}+\frac{p}{c} \biggl(1-\frac {z^{*}_{2}}{z_{n+1}} \biggr) (z_{n+1}-z_{n}) \\ =& \biggl(1-\frac {f(x^{*}_{2},y^{*}_{2},v^{*}_{2})}{f(x_{n+1},y^{*}_{2},v^{*}_{2})} \biggr) (x_{n+1}-x_{n})+ \biggl(1-\frac{y^{*}_{2}}{y_{n+1}} \biggr) (y_{n+1}-y_{n}) \\ &{} +\frac{(a+pz^{*}_{2})}{k} \biggl(v_{n+1}-v_{n}-v^{*}_{2} \ln\frac{v_{n+1}}{v_{n}} \biggr)+\frac{ay^{*}_{2} +py^{*}_{2}v^{*}_{2}}{v^{*}_{2}}(v_{n+1}-v_{n}) \phi \\ &{} -\phi\bigl(ay^{*}_{2} +py^{*}_{2}z^{*}_{2} \bigr)\ln\frac{v_{n+1}}{v_{n}}+\frac{p}{c} \biggl(1-\frac{z^{*}_{2}}{z_{n+1}} \biggr) (z_{n+1}-z_{n}). \end{aligned}$$

Substituting model (4) and $\lambda =d x^{*}_{2}+ay^{*}_{2}+py^{*}_{2}z^{*}_{2}$, we have

$$\begin{aligned} \Delta L_{n} \leq& \phi \biggl[ \biggl(1-\frac {f(x^{*}_{2},y^{*}_{2},v^{*}_{2})}{f(x_{n+1},y^{*}_{2},v^{*}_{2})} \biggr) \bigl(d x^{*}_{2}+ay^{*}_{2}+py^{*}_{2}z^{*}_{2}-d x_{n+1}-f(x_{n+1},y_{n},v_{n})v_{n} \bigr) \\ &{} + \biggl(1-\frac{y^{*}_{2}}{y_{n+1}} \biggr) \bigl(f(x_{n+1},y_{n},v_{n})v_{n}-ay_{n+1}-py_{n+1}z_{n+1} \bigr) \\ &{} +\frac{(a+pz^{*}_{2})}{k} \biggl(1-\frac {v^{*}_{2}}{v_{n+1}} \biggr) (ky_{n+1}-uv_{n+1})+\frac{ay^{*}_{2} +py^{*}_{2}v^{*}_{2}}{v^{*}_{2}}(v_{n+1}-v_{n}) \\ &{} -\bigl(ay^{*}_{2}+py^{*}_{2}z^{*}_{2} \bigr)\ln\frac{v_{n+1}}{v_{n}} +\frac{p}{c} \biggl(1-\frac{z^{*}_{2}}{z_{n+1}} \biggr) (cy_{n+1}z_{n+1}-bz_{n+1}) \biggr] \\ =& \phi \biggl[d x^{*}_{2} \biggl(1-\frac {x_{n+1}}{x^{*}_{2}} \biggr) \biggl(1-\frac {f(x^{*}_{2},y^{*}_{2},v^{*}_{2})}{f(x_{n+1},y^{*}_{2},v^{*}_{2})} \biggr) +\bigl(ay^{*}_{2}+py^{*}_{2}z^{*}_{2} \bigr) \biggl(1-\frac {f(x^{*}_{2},y^{*}_{2},v^{*}_{2})}{f(x_{n+1},y^{*}_{2},v^{*}_{2})} \\ &{} +\frac{v_{n}}{v^{*}_{2}}\frac {f(x_{n+1},y_{n},v_{n})}{f(x_{n+1},y^{*}_{2},v^{*}_{2})} \biggr) +\bigl(ay^{*}_{2}+py^{*}_{2}z^{*}_{2} \bigr) \biggl(2-\frac{v_{n}}{v^{*}_{2}} -\frac{y_{n+1}}{y^{*}_{2}}\frac{v^{*}_{2}}{v_{n+1}} \biggr) \\ &{} -\bigl(ay^{*}_{2} +py^{*}_{2}z^{*}_{2} \bigr)\frac{y^{*}_{2}}{y_{n+1}}\frac{v_{n}}{v^{*}_{2}} \frac{f(x_{n+1},y_{n},v_{n})}{f(x^{*}_{2},y^{*}_{2},v^{*}_{2})} - \bigl(ay^{*}_{2}+py^{*}_{2}z^{*}_{2} \bigr)\ln\frac{v_{n+1}}{v_{n}} \biggr] \\ =& \phi \biggl[d x^{*}_{2} \biggl(1-\frac {x_{n+1}}{x^{*}_{2}} \biggr) \biggl(1-\frac {f(x^{*}_{2},y^{*}_{2},v^{*}_{2})}{f(x_{n+1},y^{*}_{2},v^{*}_{2})} \biggr) +\bigl(ay^{*}_{2}+py^{*}_{2}z^{*}_{2} \bigr) \biggl(4-\frac{f(x^{*}_{2},y^{*}_{2},v^{*}_{2})}{f(x_{n+1},y^{*}_{2},v^{*}_{2})} \\ &{} -\frac{y^{*}_{2}}{y_{n+1}}\frac{v_{n}}{v^{*}_{2}} \frac{f(x_{n+1},y_{n},v_{n})}{f(x^{*}_{2},y^{*}_{2},v^{*}_{2})}- \frac {y_{n+1}}{y^{*}_{2}}\frac{v^{*}_{2}}{v_{n+1}} -\frac{f(x_{n+1},y^{*}_{2},v^{*}_{2})}{f(x_{n+1},y_{n},v_{n})} -\ln\frac{v_{n+1}}{v_{n}} \biggr) \\ &{} +\bigl(ay^{*}_{2}+py^{*}_{2}z^{*}_{2} \bigr) \biggl(-1-\frac{v_{n}}{v^{*}_{2}}+\frac {f(x_{n+1},y^{*}_{2},v^{*}_{2})}{f(x_{n+1},y_{n},v_{n})} +\frac {v_{n}}{v^{*}_{2}} \frac {f(x_{n+1},y_{n},v_{n})}{f(x_{n+1},y^{*}_{2},v^{*}_{2})} \biggr) \biggr]. \end{aligned}$$

Let $g(x)=x-1-\ln x$, we have

17 $$\begin{aligned}& 4-\frac {f(x^{*}_{2},y^{*}_{2},v^{*}_{2})}{f(x_{n+1},y^{*}_{2},v^{*}_{2})} -\frac{y^{*}_{2}}{y_{n+1}} \frac{v_{n}}{v^{*}_{2}} \frac{f(x_{n+1},y_{n},v_{n})}{f(x^{*}_{2},y^{*}_{2},v^{*}_{2})} \\& \quad\quad{} -\frac{y_{n+1}}{y^{*}_{2}}\frac{v^{*}_{2}}{v_{n+1}} -\frac{f(x_{n+1},y^{*}_{2},v^{*}_{2})}{f(x_{n+1},y_{n},v_{n})}-\ln \frac{v_{n+1}}{v_{n}} \\& \quad = -g\biggl(\frac {f(x^{*}_{2},y^{*}_{2},v^{*}_{2})}{f(x_{n+1},y^{*}_{2},v^{*}_{2})}\biggr) -g\biggl(\frac{y^{*}_{2}}{y_{n+1}} \frac{v_{n}}{v^{*}_{2}} \frac{f(x_{n+1},y_{n},v_{n})}{f(x^{*}_{2},y^{*}_{2},v^{*}_{2})}\biggr) \\& \quad\quad{} -g\biggl(\frac{y_{n+1}}{y^{*}_{2}}\frac{v^{*}_{2}}{v_{n+1}}\biggr) -g\biggl( \frac{f(x_{n+1},y^{*}_{2},v^{*}_{2})}{f(x_{n+1},y_{n},v_{n})}\biggr)\leq0. \end{aligned}$$

According to $(A_{2})$, we know

18 $$ \biggl(1-\frac{x_{n+1}}{x^{*}_{2}} \biggr) \biggl(1-\frac {f(x^{*}_{2},y^{*}_{2},v^{*}_{2})}{f(x_{n+1},y^{*}_{2},v^{*}_{2})} \biggr)\leq0. $$

Since $(A_{4})$ holds for $i=2$, we further obtain

19 $$ -1-\frac{v_{n}}{v^{*}_{2}}+\frac {f(x_{n+1},y^{*}_{2},v^{*}_{2})}{f(x_{n+1},y_{n},v_{n})} +\frac {v_{n}}{v^{*}_{2}} \frac {f(x_{n+1},y_{n},v_{n})}{f(x_{n+1},y^{*}_{2},v^{*}_{2})}\leq0. $$

Therefore, when $R_{1}>1$, from (17), (18), and (19) we finally have $\Delta L_{n}\leq0$. Obviously, $\Delta L_{n}=0$ if and only if $x_{n}=x^{*}_{2}$, $y_{n}=y^{*}_{2}$, $v_{n}=v^{*}_{2}$, and $z_{n}=z^{*}_{2}$. Based on LaSalle’s invariance principle, we finally see that the infected equilibrium $E_{2}(x^{*}_{2},y^{*}_{2},v^{*}_{2},z^{*}_{2})$ is globally asymptotically stable. This completes the proof. □

As a consequence of Theorem 5 we have the following result for model (5).

### Corollary 3

*Let* $R_{1}>1$. *Then the infected equilibrium* $E_{2}(x^{*}_{2},y^{*}_{2},v^{*}_{2},z^{*}_{2})$ *of model* (5) *is globally asymptotically stable*.

## Numerical examples

In this section, we give the numerical examples to discuss assumption $(A_{4})$. In model (4), we choose a nonlinear incidence $f(x,y,v)=\frac{\beta x}{1+nv^{2}}$. Furthermore, $h=1$ in the denominator function *ϕ*. The mortality rate of the CTL response *b* in model (4) is chosen as a free parameter. All remaining parameters in model (4) are chosen as in Table 1.

**Table 1:** **List of parameters**

Parameter	Definition	Value	Source
*λ*	Production rate of uninfected cells	10	References [31, 32]
*d*	Death rate of uninfected cells	0.1	References [31, 32]
*β*	Infection rate	0.15	References [31, 32]
*a*	Death rate of infected cells	0.2	References [19, 31]
*p*	CTL effectiveness	1	References [19, 31]
*n*	Saturation coefficient	0.01	Reference [7]
*k*	Production rate of free virus	0.1	References [19, 31]
*u*	Clearance rate of free virus	0.1	References [19, 30]
*c*	Proliferation rate of CTL response	0.01	References [19, 30]

We first take the mortality rate of CTL response $b=0.75$. By calculating, we see that the basic reproduction numbers $R_{0}\doteq 75>1$ and $R_{1}\doteq0.5216<1$. Furthermore, we also have $\frac {\lambda}{\xi}=100$. Hence, model (4) has only the virus-free equilibrium $E_{0}(100,0,0,0,)$ and the no-immune equilibrium $E_{1}(21.745,39.127,39.127,0)$.

Consider assumption $(A_{4})$. By calculating we obtain

20 $$ \biggl(1-\frac{f(x,y,v)}{f(x,y^{*}_{i},v^{*}_{i})}\biggr) \biggl(\frac{f(x,y^{*}_{i},v^{*}_{i})}{f(x,y,v)}- \frac{v}{v^{*}_{i}}\biggr) =\biggl(1-\frac{1+nv^{*2}_{i}}{1+nv^{2}}\biggr) (v-v^{*}_{i}) (nvv^{*}_{i}-1). $$

For $i=1$, since $n\frac{\lambda}{\xi}v^{*}_{1}-1\doteq38.127>0$, where $v^{*}_{1}\doteq39.127$, from (20) we see that assumption $(A_{4})$ for $i=1$ is not satisfied.

**Figure 1:**
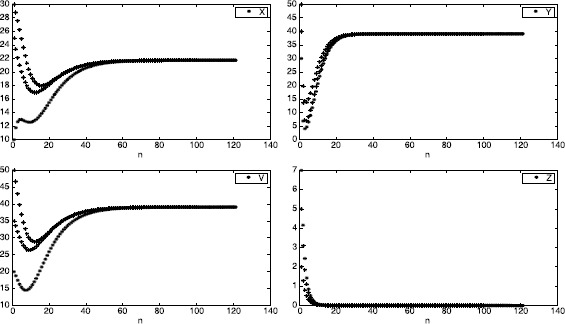
**The trajectories of solutions** $\pmb{(x_{n},y_{n},v_{n},z_{n})}$ **with initial values** $\pmb{(x_{0},y_{0},v_{0},z_{0})=(10,30,20,7), (25,40,35,2),\text{ and }(30,50,50,5)}$ **.**

However, the numerical simulations given in Figure 1 show that equilibrium $E_{1}$ is globally asymptotically stable.

We next take the mortality rate of CTL response $b=0.15$. By calculating, we see that the basic reproduction numbers $R_{0}\doteq 75>1$ and $R_{1}\doteq2.608>1$. Furthermore, we also have $\frac {\lambda}{\xi}=100$. Hence, model (4) has the virus-free equilibrium $E_{0}(100,0,0,0)$, the no-immune equilibrium $E_{1}(21.746,39.127,39.127,0)$, and the infected equilibrium $E_{2}(12.621,15,15,0.383)$.

Consider assumption $(A_{4})$. Since $n\frac{\lambda}{\xi}v^{*}_{2}-1\doteq 14>0$, where $v^{*}_{2}\doteq15$, from (20) we see that assumption $(A_{4})$ for $i=2$ is not satisfied.

**Figure 2:**
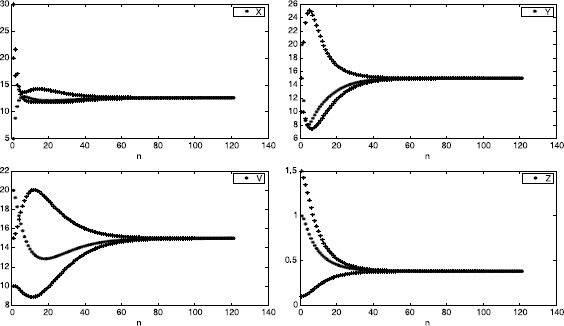
**The trajectories of solutions** $\pmb{(x_{n},y_{n},v_{n},z_{n})}$ **with initial values** $\pmb{(x_{0},y_{0},v_{0},z_{0})=(20,15,15,0.1), (30,10,10,1.5),\text{ and }(5,20,20,1)}$ **.**

However, the numerical simulations given in Figure 2 show that equilibrium $E_{2}$ is globally asymptotically stable.

The above numerical examples show that even if assumption $(A_{4})$ does not hold, the no-immune equilibrium may be globally asymptotically stable only when $R_{0}>1$ and $R_{1}<1$, and the infected equilibrium may be globally asymptotically stable only when $R_{0}>1$ and $R_{1}>1$.

## Discussions

In this paper, we studied a four dimensional discrete-time virus infected model (4) with general nonlinear incidence function $f(x,y,v)v$ and CTL immune response obeying Micken’s non-standard finite difference (NSFD) scheme. Assumptions $(A_{1})$-$(A_{4})$ for nonlinear function $f(x,y,v)$ are introduced and two basic reproduction numbers $R_{0}$ and $R_{1}$ also are defined. The basic properties of model (4) on the existence of the virus-free equilibrium $E_{0}$, the no-immune equilibrium $E_{1}$, and the infected equilibrium $E_{2}$, and the positivity and ultimate boundedness of the solutions are established. Under $(A_{1})$-$(A_{4})$, the global stability and instability of the equilibria are completely determined by the basic reproduction numbers $R_{0}$ and $R_{1}$. That is, if $R_{0}\leq1$ then $E_{0}$ is globally asymptotically stable, if $R_{0}>1$ and $R_{1}\leq1$ then $E_{0}$ is unstable and $E_{1}$ is globally asymptotically stable and if $R_{0}>1$ and $R_{1}>1$ then $E_{0}$ and $E_{1}$ are unstable and $E_{2}$ is globally asymptotically stable.

We see that $(A_{1})$-$(A_{3})$ are basic for model (4). Particularly, when $f(x,y,v)=\frac{\beta x}{1+mx+nv}$ and $f(x,y,v)=\frac{\beta x}{1+nv^{q}}$ then $(A_{1})$-$(A_{3})$ naturally hold. But $(A_{4})$ is a mathematical assumption. It is only used in the proofs of theorems on the global stability of the no-immune equilibrium $E_{1}$ and the infected equilibrium $E_{2}$ to obtain $\Delta L_{n}\leq0$ for the Lyapunov function $L_{n}$ (see the proofs of Theorem 4 and Theorem 5). However, we also see that when $f(x,y,v)=\frac{\beta x}{1+mx+nv}$, $(A_{4})$ naturally hold. Furthermore, the numerical simulations given in Section 4 show that even if $(A_{4})$ does not hold, the no-immune equilibrium $E_{1}$ may be globally asymptotically stable only when $R_{0}>1$ and $R_{1}<1$, and the infected equilibrium $E_{2}$ may be globally asymptotically stable only when $R_{0}>1$ and $R_{1}>1$.

Generally, we expect that the global stability of the equilibria for model (4) can be completely determined only by the basic reproduction numbers $R_{0}$ and $R_{1}$. Therefore, an open problem is whether $(A_{4})$ can be thrown off in Theorem 4 and Theorem 5. Furthermore, we also do not obtain the local asymptotic stability of the infected equilibrium $E_{2}$ only under $(A_{1})$-$(A_{3})$. The cause is that the characteristic equation of linearized system of model (4) at equilibrium $E_{2}$ is very complicated.

When the incidence function $f(x,y,v)=\frac{\beta x}{1+mx+nv}$, we know that $(A_{1})$-$(A_{4})$ are satisfied. The global stability of the equilibria of the discrete model (5) only depends on the basic reproduction numbers $R_{0}$ and $R_{1}$. This shows that the global stability of the equilibria for the discrete model (5) is equal to the corresponding continuous model (3). This implies that the NSFD scheme preserves the stability of the continuous model.

As is well known, in our body the immune response is made up of both a cellular response and a humoral response. The cellular response is that T cells kill the infected cells, the humoral response is that B cells produce an antibody to neutralize the virus. In this paper, we only consider the cellular response. In the future, our work will focus on the idea that the two kinds of immune response simultaneously play a role.

## References

[1] Hattaf K, Yousfi N, Tridane A (2012). Mathematical analysis of a virus dynamics model with general incidence rate and cure rate. Nonlinear Anal., Real World Appl..

[2] Wang X, Tao Y, Song X (2011). Global stability of a virus dynamics model with Beddington-DeAngelis incidence rate and CTL immune response. Nonlinear Dyn..

[3] Hattaf K, Khabouze M, Yousfi N (2014). Dynamics of a generalized viral infection model with adaptive immune response. Int. J. Dyn. Control.

[4] Yan Y, Wang W (2012). Global stability of a five-dimensional model with immune responses and delay. Discrete Contin. Dyn. Syst., Ser. B.

[5] Korobeinikov A (2004). Global properties of basic virus dynamics models. Bull. Math. Biol..

[6] Li MY, Shu H (2010). Global dynamics of an in-host viral model with intracellular delay. Bull. Math. Biol..

[7] Balasubramaniam P, Tamilalagan P, Prakash M (2015). Bifurcation analysis of HIV infection model with antibody and cytotoxic T-lymphocyte immune responses and Beddington-DeAngelis functional response. Math. Methods Appl. Sci..

[8] Tian Y, Liu X (2014). Global dynamics of a virus dynamical model with general incidence rate and cure rate. Nonlinear Anal., Real World Appl..

[9] Yousfi N, Hattaf K, Tridane A (2011). Modeling the adaptive immune response in HBV infection. J. Math. Biol..

[10] Hattaf K, Yousfi N, Tridane A (2013). Stability analysis of a virus dynamics model with general incidence rate and two delays. Appl. Math. Comput..

[11] Lu X, Hui L, Liu S, Li J (2015). A mathematical model of HIV-I infection with two time delays. Math. Biosci. Eng..

[12] Hattaf K, Yousfi N, Tridane A (2014). A delay virus dynamics model with general incidence rate. Differ. Equ. Dyn. Syst..

[13] Zhu H, Luo Y, Chen M (2011). Stability and Hopf bifurcation of a HIV infection model with CTL-response delay. Comput. Math. Appl..

[14] Hu Z, Zhang J, Wang H, Ma W, Liao F (2014). Dynamics analysis of a delayed viral infection model with logistic growth and immune impairment. Appl. Math. Model..

[15] Wang T, Hu Z, Liao F (2014). Stability and Hopf bifurcation for a virus infection model with delayed humoral immunity response. J. Math. Anal. Appl..

[16] Shi X, Zhou X, Song X (2010). Dynamical behaviors of a delay virus dynamics model with CTL immune response. Nonlinear Anal., Real World Appl..

[17] Zhu H, Zou X (2008). Impact of delays in cell infection and virus production on HIV-1 dynamics. Math. Med. Biol..

[18] Pawelek KA, Liu S, Pahlevani F, Rong L (2012). A model of HIV-1 infection with two time delays: mathematical analysis and comparison with patient data. Math. Biosci..

[19] Wang Z, Xu R (2012). Stability and Hopf bifurcation in a viral infection model with nonlinear incidence rate and delayed immune response. Commun. Nonlinear Sci. Numer. Simul..

[20] Huang G, Ma W, Takeuchi Y (2009). Global properties for virus dynamics model with Beddington-DeAngelis functional response. Appl. Math. Lett..

[21] Wang K, Wang W, Pang H, Liu X (2007). Complex dynamic behavior in a viral model with delayed immune response. Physica D.

[22] Shi P, Dong L (2014). Dynamical behaviors of discrete HIV-1 virus model with bilinear infective rate. Math. Methods Appl. Sci..

[23] Wodarz D (2003). Hepatitis C virus dynamics and pathology: the role of CTL and antibody response. J. Gen. Virol..

[24] Wang Y, Zhou Y, Brauer F, Heffernan JM (2013). Viral dynamics model with CTL immune response incorporating antiretroviral therapy. J. Math. Biol..

[25] Stafford M, Corey L, Cao Y, Daar E, Ho D, Perelson A (2000). Modelling plasma virus concentration during primary HIV infection. J. Theor. Biol..

[26] Hattaf K, Yousfi N (2016). Global properties of a discrete viral infection model with general incidence rate. Math. Methods Appl. Sci..

[27] Hattaf K, Lashari AA, Boukari BE, Yousfi N (2015). Effect of discretization on dynamical behavior in an epidemiological model. Differ. Equ. Dyn. Syst..

[28] Hattaf K, Yousfi N (2015). A numerical method for a delayed viral infection model with general incidence rate. J. King Saud Univ., Sci..

[29] Mickens RE (2000). Application of Nonstandard Finite Difference Scheme.

[30] Mickens RE (2007). Calculation of denominator functions for nonstandard finite difference schemes for differential equations satisfying a positivity condition. Numer. Methods Partial Differ. Equ..

[31] Mickens RE (2003). Dynamics consistency: a fundamental principle for constructing nonstandard finite difference scheme for differential equation. J. Differ. Equ. Appl..

[32] Mickens RE, Washington T (2012). A note on an NSFD scheme for a mathematical model of respiratory virus transmission. J. Differ. Equ. Appl..

[33] LaSalle JP (1976). The Stability of Dynamical Systems.

